# A Dictionary of Medical Science; Containing a Concise Explanation of the Various Subjects and Terms; with the French and Other Synonymes; Notices of Climate, and of Celebrated Mineral Waters; Formulæ for Various Officinal and Empirical Preparations Etc.

**Published:** 1848-10

**Authors:** 


*A Dictionary of Medical Science; containing a Concise Explanation of the various Subjects and Terms; with the French and other Synonymes; Notices of Climate, and of Celebrated Mineral Waters; Formulæ for various Officinal and Empirical Preparations, etc.* By Robley Dunglison, M.D., Professor of the Institutes of Medicine in Jefferson Medical College, Philadelphia. Seventh edition, carefully revised and greatly enlarged. Royal octavo. pp. 912.

The late hour at which this important work has been issued from the press, (Sept. 1848,) just enables us to announce its appearance, without criticism or extended notice. That a work of this description should have gone to the seventh edition, (and *large* editions too,) in the course of a very few years, is as remarkable as it is creditable to the liberality of the medical profession in the United States, and must be exceedingly gratifying to the gifted and laborious author.

Some idea of the extent and completeness of this Dictionary may be gained from the fact stated by the publishers, that it now contains " *over forty-five thousand words;*" and of the labour and pains bestowed by the author in the preparation of each succeeding edition, from the circumstance that the last or sixth edition, comprised two thousand five hundred subjects and terms not contained in the preceding, and that there are in the present *over six thousand terms* not contained in the last edition! How different is the work from what it would have been, if the author, to save labour, and the publishers, to spare expense, had chosen to issue it from stereotype plates. By the course which is pursued the book keeps pace with science, and the reader, in looking into its pages for a new and obscure term, is almost certain to find it, which is the great charm of a good dictionary.

